# The association of the Activities of Daily Living and the outcome of old intensive care patients suffering from COVID-19

**DOI:** 10.1186/s13613-022-00996-9

**Published:** 2022-03-18

**Authors:** Raphael Romano Bruno, Bernhard Wernly, Hans Flaatten, Jesper Fjølner, Antonio Artigas, Philipp Heinrich Baldia, Stephan Binneboessel, Bernardo Bollen Pinto, Joerg C. Schefold, Georg Wolff, Malte Kelm, Michael Beil, Sigal Sviri, Peter Vernon van Heerden, Wojciech Szczeklik, Muhammed Elhadi, Michael Joannidis, Sandra Oeyen, Eumorfia Kondili, Brian Marsh, Jakob Wollborn, Finn H. Andersen, Rui Moreno, Susannah Leaver, Ariane Boumendil, Dylan W. De Lange, Bertrand Guidet, Christian Jung, COVIP study group, Philipp Eller, Michael Joannidis, Dieter Mesotten, Pascal Reper, Sandra Oeyen, Walter Swinnen, Nicolas Serck, Elisabeth Dewaele, Helene Brix, Jens Brushoej, Pritpal Kumar, Helene Korvenius Nedergaard, Ida Riise Balleby, Camilla Bundesen, Maria Aagaard Hansen, Stine Uhrenholt, Helle Bundgaard, Jesper Fjølner, James Gooch, Lenka Cagova, Elizabeth Potter, Michael Reay, Miriam Davey, Mohammed Abdelshafy Abusayed, Sally Humphreys, Arnaud Galbois, Bertrand Guidet, Cyril Charron, Caroline Hauw Berlemont, Guillaume Besch, Jean-Philippe Rigaud, Julien Maizel, Michel Djibré, Philippe Burtin, Pierre Garcon, Saad Nseir, Xavier Valette, Nica Alexandru, Nathalie Marin, Marie Vaissiere, Gaëtan Plantefeve, Thierry Vanderlinden, Igor Jurcisin, Buno Megarbane, Benjamin Glenn Chousterman, François Dépret, Marc Garnier, Sebastien Besset, Johanna Oziel, Alexis Ferre, Stéphane Dauger, Guillaume Dumas, Bruno Goncalves, Lucie Vettoretti, Didier Thevenin, Stefan Schaller, Muhammed Kurt, Andreas Faltlhauser, Christian Meyer, Milena Milovanovic, Matthias Lutz, Gonxhe Shala, Hendrik Haake, Winfried Randerath, Anselm Kunstein, Patrick Meybohm, Stephan Steiner, Eberhard Barth, Tudor Poerner, Philipp Simon, Marco Lorenz, Zouhir Dindane, Karl Friedrich Kuhn, Martin Welte, Ingo Voigt, Hans-Joachim Kabitz, Jakob Wollborn, Ulrich Goebel, Sandra Emily Stoll, Detlef Kindgen-Milles, Simon Dubler, Christian Jung, Kristina Fuest, Michael Schuster, Antonios Papadogoulas, Francesk Mulita, Nikoletta Rovina, Zoi Aidoni, Evangelia Chrisanthopoulou, Eumorfia Kondili, Ioannis Andrianopoulos, Martijn Groenendijk, Mirjam Evers, Lenneke van Lelyveld-Haas, Iwan Meynaar, Alexander Daniel Cornet, Marieke Zegers, Willem Dieperink, Dylan W. De Lange, Tom Dormans, Michael Hahn, Britt Sjøbøe, Hans Frank Strietzel, Theresa Olasveengen, Luis Romundstad, Finn H. Andersen, Anna Kluzik, Paweł Zatorski, Tomasz Drygalski, Wojciech Szczeklik, Jakub Klimkiewicz, Joanna Solek-pastuszka, Dariusz Onichimowski, Miroslaw Czuczwar, Ryszard Gawda, Jan Stefaniak, Karina Stefanska-Wronka, Ewa Zabul, Ana Isabel Pinho Oliveira, Rui Assis, Maria de Lurdes Campos Santos, Henrique Santos, Filipe Sousa Cardoso, André Gordinho, Maria José Arche Banzo, Begoña Zalba-Etayo, Patricia Patricia Cubero, Jesús Priego, Gemma Gomà, Teresa Maria Tomasa-Irriguible, Susana Sancho, Aida Fernández Ferreira, Eric Mayor Vázquez, Ángela Prado Mira, Mercedes Ibarz, David Iglesias, Susana Arias-Rivera, Fernando Frutos-Vivar, Sonia Lopez-Cuenca, Cesar Aldecoa, David Perez-Torres, Isabel Canas-Perez, Luis Tamayo-Lomas, Cristina Diaz-Rodriguez, Pablo Ruiz de Gopegui, Nawfel Ben-Hamouda, Andrea Roberti, Yvan Fleury, Nour Abidi, Joerg C. Schefold, Ivan Chau, Alexander Dullenkopf, Richard Pugh, Sara Smuts

**Affiliations:** 1 Medical Faculty, Department of Cardiology, Pulmonology and Vascular Medicine, Heinrich-Heine-University Duesseldorf, Moorenstraße 5, 40225 Duesseldorf, Germany; 2 Department of Internal Medicine, General Hospital Oberndorf, Teaching Hospital of the Paracelsus Medical University Salzburg, Paracelsusstraße 37, Oberndorf, 5110 Salzburg, Austria; 3 Center for Public Health and Healthcare Research, Paracelsus Medical University Salzburg, 5020 Salzburg, Austria; 4 Department of Clinical Medicine, Department of Anaestesia and Intensive Care, Haukeland University Hospital, University of Bergen, Bergen, Norway; 5 Department of Intensive Care, Aarhus University Hospital, Aarhus, Denmark; 6 Department of Intensive Care Medicine, CIBER Enfermedades Respiratorias, Corporacion Sanitaria Universitaria Parc Tauli, Autonomous University of Barcelona, Sabadell, Spain; 7 Department of Acute Medicine, Geneva University Hospitals, Geneva, Switzerland; 8 Department of Intensive Care Medicine, Inselspital, Universitätsspital, University of Bern, Bern, Switzerland; 9 Department of Medical Intensive Care, Hadassah Medical Center and Faculty of Medicine, Hebrew University of Jerusalem, Jerusalem, Israel; 10 Department of Intensive Care and Perioperative Medicine, Jagiellonian University Medical College, Krakow, Poland; 11 Faculty of Medicine, University of Tripoli, Tripoli, Libya; 12 Division of Intensive Care and Emergency Medicine, Department of Internal Medicine, Medical University Innsbruck, Innsbruck, Austria; 13 Department of Intensive Care 1K12IC, Ghent University Hospital, Ghent, Belgium; 14 Intensive Care Unit, University Hospital of Heraklion, Heraklion, Greece; 15 Mater Misericordiae University Hospital, Dublin, Ireland; 16 Department of Anesthesiolgy, Perioperative and Pain Medicine, Brigham and Women’s Hospital, Harvard Medical School, Boston, USA; 17 Department of Anaesthesia and Intensive Care, Ålesund Hospital, Ålesund, Norway; 18 Department of Circulation and Medical Imaging, Norwegian University of Science and Technology, Trondheim, Norway; 19 Hospital de São José, Centro Hospitalar Universitário de Lisboa Central, Faculdade de Ciências Médicas de Lisboa, Nova Médical School, Lisbon, Portugal; 20 Universidade da Beira Interior, Covilhã, Portugal; 21 General Intensive Care, St George´S University Hospitals NHS Foundation Trust, London, UK; 22 Sorbonne Universités, UPMC Univ Paris 06, INSERM, UMR_S 1136, Institut Pierre Louis d’Epidémiologie et de Santé Publique, Equipe: épidémiologie hospitalière qualité et organisation des soins, 75012 Paris, France; 23 Assistance Publique - Hôpitaux de Paris, Hôpital Saint-Antoine, Service de réanimation médicale, 75012 Paris, France; 24 Department of Intensive Care Medicine, University Medical Center, University Utrecht, Utrecht, the Netherlands

## Abstract

**Purpose:**

Critically ill old intensive care unit (ICU) patients suffering from Sars-CoV-2 disease (COVID-19) are at increased risk for adverse outcomes. This post hoc analysis investigates the association of the Activities of Daily Living (ADL) with the outcome in this vulnerable patient group.

**Methods:**

The COVIP study is a prospective international observational study that recruited ICU patients ≥ 70 years admitted with COVID-19 (NCT04321265). Several parameters including ADL (ADL; 0 = disability, 6 = no disability), Clinical Frailty Scale (CFS), SOFA score, intensive care treatment, ICU- and 3-month survival were recorded. A mixed-effects Weibull proportional hazard regression analyses for 3-month mortality adjusted for multiple confounders.

**Results:**

This pre-specified analysis included 2359 patients with a documented ADL and CFS. Most patients evidenced independence in their daily living before hospital admission (80% with ADL = 6). Patients with no frailty and no disability showed the lowest, patients with frailty (CFS ≥ 5) and disability (ADL < 6) the highest 3-month mortality (52 vs. 78%, *p* < 0.001). ADL was independently associated with 3-month mortality (ADL as a continuous variable: aHR 0.88 (95% CI 0.82–0.94, *p* < 0.001). Being “disable” resulted in a significant increased risk for 3-month mortality (aHR 1.53 (95% CI 1.19–1.97, *p* 0.001) even after adjustment for multiple confounders.

**Conclusion:**

Baseline Activities of Daily Living (ADL) on admission provides additional information for outcome prediction, although most critically ill old intensive care patients suffering from COVID-19 had no restriction in their ADL prior to ICU admission. Combining frailty and disability identifies a subgroup with particularly high mortality.

*Trial registration number:* NCT04321265.

**Graphical Abstract:**

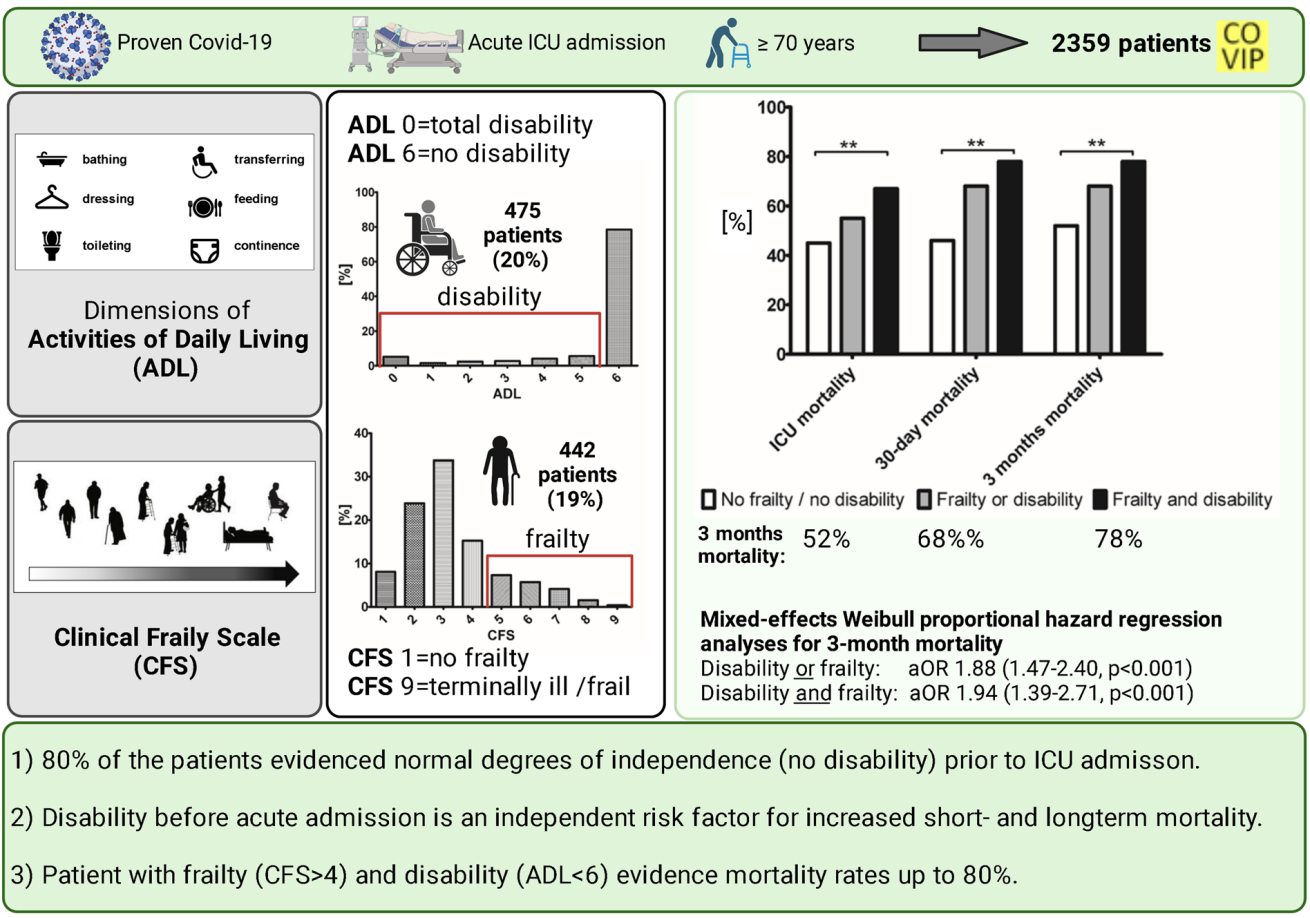

**Supplementary Information:**

The online version contains supplementary material available at 10.1186/s13613-022-00996-9.

## Introduction

Older patients admitted to an intensive care unit are at significantly increased risk for adverse outcome [1, 2]. Old patients make up the subgroup of intensive care unit patients with the highest mortality [3]. However, the chronological age is a worse parameter for the outcome prediction of critically ill older patients [4, 5]. This is particularly true for SARS-CoV-2 and its disease COVID-19, which challenge intensive care units worldwide [6]. During the peaks of pandemic, the sheer volume of patients with COVID-19 has overwhelmed intensive care resources in many hospitals. In some countries, age cut-offs for ICU admission had been discussed [7], but soon medical societies recommend other instruments for triage and outcome prediction. However, the evidence for some components of the triage decision-making was relatively scarce. A well-established tool is the Clinical Frailty Scale (CFS) which assesses the functioning of the old patient regarding fitness and frailty. Its association with ICU and 30-day mortality showed its importance for outcome prediction with and without Covid-19 [1, 2, 6, 8]. Apart from CFS, other instruments have been proposed. The Israeli Position Paper, for example, named the Activities of Daily Living (ADL) as a candidate for the medical assessment tools that should be considered for assessing function during triage situations [9]. ADL had been introduced in the early 1980s. It is a tool to evaluate individual independence in daily living. Initially, it was intended to assess the performance of older patients determining life expectancy [10]. The scale includes routine tasks, which patients perform during their daily routines, such as basic feeding, bathing, movement (transferring and getting out of bed), sphincter control, and bathroom use. For non-COVID patients, it has been found that old patients requiring mechanical ventilation, low ADL scores—meaning higher grades of dependence—were independently associated with worse outcome [11]. Currently, the value of ADL for outcome prediction of severe COVID-19 and its sequelae remains unclear. To address this lack of evidence, we performed a post hoc analysis of the COVIP-Study (COVID-19 in very old intensive care patients).

This multicentre study investigates the association of pre-existing disability regarding the Activities of Daily Living (ADL) with and without pre-existing frailty (CFS) on the one hand and the outcome on the other hand in a large prospectively recruited cohort of old ICU patients (≥ 70 years) with COVID-19.

## Methods

### Design and settings

COVIP aimed to identify predictors for mortality in older patients suffering from severe COVID-19. This multicentre study was part of the Very old Intensive care Patients (VIP) project and was endorsed by the European Society of Intensive Care Medicine (ESICM) (www.vipstudy.org). COVIP was registered on ClinicalTrials.gov (ID: NCT04321265) and followed the European Union General Data Privacy Regulation (GDPR) directive applied in most participating countries. As in the previous VIP-studies [1, 2], national coordinators recruited the ICUs, coordinated national and local ethical permissions, and supervised patient recruitment at national level. For all centres ethical approval was mandatory for study participation. In most countries, informed consent was obligatory for inclusion depending on local legal regulations. This study used patient data from 151 ICUs from 26 independent countries, including European ICUs, and the Asian, African, and Americas.

### Study population

Patients with proven (PCR diagnosed) COVID-19 aged 70 years or older who were admitted to an intensive care unit were recruited. The dataset for this subgroup analysis was extracted from the study database on the 15th of July 2021. Thus, the database included patients who were admitted from the 19th of March 2020 to the 15th of July 2021. Data collection for each patient commenced at ICU admission. The admission day was defined as day one, and all consecutive days were numbered sequentially from the admission date. For this analysis, only patients with a documented CFS and ADL were included.

### Data collection and storage

All centres used a uniform online electronic case report form (eCRF). As previously analysis, only patients with a documented ADL were included. As previously, the Sequential Organ Failure Assessment (SOFA) score on admission was calculated either manually or using an online calculator in the eCRF [1, 2]. Furthermore, COVIP assessed the need for non-invasive or invasive ventilation, prone positioning, tracheostomy, vasopressor use and renal replacement therapy. The eCRF also asked about any limitation of life-sustaining treatment during the ICU stay. The eCRF and database ran on a secure server composed and stored in Aarhus University, Denmark.

### The activity of daily living, frailty, and comorbidities

The Katz Activities of Daily Living (ADL) score assessed the patient's independence in daily living before hospital admission. ADL is a commonly utilised graded instrument to evaluate disability and the level of dependence in chronically ill or older patients. It assesses six primary and psychosocial functions: bathing, dressing, going to the toilet, transferring, feeding, and continence. Every patient receives 1 point for each independent and 0 for every dependent activity (6 = independent patient, 0 = very dependent patient). Depending on the trial and context, the cut-off defining “disability” varies. ADL could be obtained by the patient himself, by caregivers/family, hospital records, or other sources. To characterise the cohort more precisely, we divided it into two groups: an ADL score of 6 was defined as “no disability”, < 6 as “disable” [12]. The Clinical Frailty Scale (CFS) was evaluated as described previously [1, 2]. The respective visual and simple description for this assessment tool was used with permission [10, 13, 14] and distinguished nine classes of frailty from very fit (CFS 1) to terminally ill (CFS 9). A CFS ≥ 5 was considered as “frailty”. The SOFA score was recorded on admission; it was calculated manually or using an online calculator. In the next step, patients were divided into three groups according to their CFS and ADL: Patients without frailty and without disability (CFS < 5, ADL 6), patients with either frailty or disability CFS ≥ 5 or ADL < 6, and patients with both frailty and disability (CFS ≥ 5 and ADL < 6).

### Statistical analysis

We did not perform a formal sample size calculation prior to this purely observational study. The analysis plan was finalised prior to any analysis. The primary exposure were disability (ADL) and frailty (CFS), the primary outcome was 30-month survival, and the secondary outcomes were overall survival up to discharge from ICU, survival 30 days after ICU admission, organ support (vasoactive drugs, invasive mechanical ventilation, non-invasive ventilation, and renal replacement therapy) and treatment limitation. Continuous data points are expressed as median ± interquartile range. Differences between independent groups were calculated using the Mann–Whitney U-test. Categorical data are expressed as numbers (percentage). The Chi-square test was applied to calculate differences between groups. A mixed-effects Weibull proportional hazard regression was performed using ADL as a categorical (ADL 6 or ADL ≥ 5 meaning a patient with independence in daily living) and continuous variable (ADL 0 to 6) and 3-month mortality (primary outcome). We fitted models for the dependent variables with robust standard errors. The regression analyses were conducted using only robust estimators of the standard errors and not in the sense of robustness against violations of normality assumptions as for the robust methods (e.g., Mann–Whitney tests) used for the univariate analyses [15]. Three models was performed [16]. Model-2 added age, gender und SOFA. To adjust the effect for ICU capacities and COVID-19 incidence, model-3 additionally comprises ICU beds per 100.000 per country and the local COVID incidence on the day of ICU admission. We chose the independent variables based on differences in the baseline characteristics, previous reports, and our own clinical experience. The adjusted hazard ratios (HR) with respective 95% confidence intervals (95% CI) were calculated: HR describes the change in risk of death per each unit increase for continuous variables and for one specific category vs. a reference category for categorical variables. A HR > 1 suggests an increase in the risk of death, HR < 1 suggest a decrease in the risk of death. All tests were two-sided, and a p-value of < 0.05 was considered statistically significant. Since not all parameters were available for all categories, patients had to be excluded for the subgroup analyses. For this reason, not all patient numbers add up to 100% (see Tables 1, 2, 3, and 4). Stata 16 was used for all statistical computations (StataCorp LLC, 4905 Lakeway Drive, College Station, Brownsville, Texas, USA). GraphPad Prism 5 (GraphPad Software, San Diego, CA 92108, USA) was used for figures.

**Table 1:** Baseline characteristics of patients with disability (ADL < 6) and without (ADL 6)

	No disability (ADL 6)	Disability (ADL < 6)	*p*-value
	*N* = 1884	*N* = 475	
Male sex ([%], *n*)	74% (1,394)	58% (276)	< 0.001
Age (years)	75 (4)	78 (5)	< 0.001
SOFA	5 (3)	7 (4)	< 0.001
CFS	3 (1)	5 (2)	< 0.001
Diabetes mellitus ([%], *n*)	31% (589)	52% (246)	< 0.001
CAD ([%], *n*)	20% (380)	34% (160)	< 0.001
Chronic renal failure ([%], *n*)	12% (230)	29% (139)	< 0.001
Arterial hypertension ([%], *n*)	64% (1,212)	77% (364)	< 0.001
Pulmonary disease ([%], *n*)	21% (398)	32% (151)	< 0.001
Chronic heart failure ([%], *n*)	12% (219)	28% (133)	< 0.001

*ADL* Activities of Daily Living, *CFS* Clinical Frailty Scale, *CAD* coronary artery disease, *SOFA* Sequential Organ Failure Assessment; *p*-value comparing all groups. [Numbers do not add up to 100% due to missing values]

**Table 2:** Baseline characteristics of patients without frailty (CFS < 5) and disability (ADL 6), patients with either disability (ADL < 6) or Frailty (CFS ≥ 5), or both frailty and disability

	Non-frailty (CFS < 5)/no disability (ADL 6)	Frailty (CFS ≥ 5) or disability (ADL < 6)	Frailty and disability	*p*-value
	*n* = 1829	*n* = 260	*n* = 270	
Male sex ([%], *n*)	73% (1342)	67% (173)	57% (155)	< 0.001
Age (years)	75 (4)	77 (6)	78 (5)	< 0.001
SOFA	5 (3)	6 (3)	8 (4)	< 0.001
CFS	3 (1)	5 (1)	6 (1)	< 0.001
Diabetes mellitus ([%], *n*)	31% (557)	48% (125)	57% (153)	< 0.001
CAD ([%], *n*)	20% (358)	30% (78)	39% (104)	< 0.001
Chronic renal failure ([%], *n*)	11% (206)	27% (71)	34% (92)	< 0.001
Arterial hypertension ([%], *n*)	64% (1167)	75% (194)	80% (215)	< 0.001
Pulmonary disease ([%], *n*)	21% (377)	29% (74)	36% (98)	< 0.001
Chronic heart failure ([%], *n*)	11% (196)	29% (73)	31% (83)	< 0.001

*ADL* Activities of Daily Living, *CFS* Clinical Frailty Scale, *CAD* coronary artery disease, *SOFA* Sequential Organ Failure Assessment, *p*-value comparing all groups. [Numbers do not add up to 100% due to missing values]

**Table 3:** Outcome and intensive care treatment of patients without frailty (CFS < 5) and disability (ADL 6), patients with either disability (ADL < 6) or frailty (CFS ≥ 5), or both frailty and disability

	Non-frailty (CFS < 5)/no disability (ADL 6)	Frailty (CFS ≥ 5) or disability (ADL < 6)	Frailty and disability	*p*-value
Invasive mechanical ventilation	76% (1388)	62% (162)	68% (183)	< 0.001
Non-invasive mechanical ventilation	23% (414)	29% (75)	31% (84)	0.002
Tracheostomy	21% (376)	12% (31)	10% (28)	< 0.001
Vasoactive drugs	72% (1312)	58% (149)	67% (178)	< 0.001
RRT	14% (255)	16% (41)	23% (61)	< 0.001
Life sustaining care withheld	30% (540)	42% (106)	31% (83)	< 0.001
Life sustaining care withdrawn	20% (369)	21% (52)	16% (44)	0.32
ICU mortality	45% (810)	55% (140)	67% (180)	< 0.001
30-day mortality	46% (848)	63% (162)	72% (195)	< 0.001
3-month mortality	52% (945)	68% (176)	78% (210)	< 0.001

*ADL* Activities of Daily Living, *CFS* Clinical Frailty Scale, *RRT* renal replacement therapy, *p*-value comparing all groups. [Numbers do not add up to 100% due to missing values]

**Table 4:** Mixed-effects Weibull proportional hazard regression analyses for 3-month mortality (aHR (95% CI, *p*-value))

	Model-1	Model-2	Model-3
ADL continuous	0.84 (0.80–0.89, *p* < 0.001)	0.91 (0.87–0.96, *p* 0.001)	0.88 (0.82–0.94, *p* < 0.001)
ADL binary (ADL < 6)	1.83 (1.50–2.21, *p* < 0.001)	1.34 (1.09–1.65, *p* 0.006)	1.53 (1.19–1.97, *p* 0.001)
ADL binary (ADL < 5)	1.75 (1.44–2.13, *p* < 0.001)	1.23 (0.99–1.52, *p* 0.060)	1.57 (1.21–2.03, *p* 0.001)
Frailty or disability (ADL < 6 or CFS ≥ 5)	1.77 (1.39–2.25, *p* < 0.001)	1.51 (1.22–1.88, *p* < 0.001)	1.88 (1.47–2.40, *p* < 0.001)
Frailty and disability	2.43 (1.87–3.16, *p* < 0.001)	1.58 (1.19–2.10, *p* 0.002)	1.94 (1.39–2.71, *p* < 0.001)

*aHR* adjusted hazard ratio, *ADL* Activities of Daily Living, *CFS* Clinical Frailty Scale, *ICU* intensive care unit, *SOFA* Sequential Organ Failure Assessment

Model-1: Individual ICU as random effect, and ADL/CFS as fixed effects

Model-2: Model-1 plus SOFA, gender, age

Model-3: Model-2 plus ICU beds per 100.000 per country and the local COVID-19 incidence on the day of ICU admission

## Results

### Study population

**Fig. 1:**
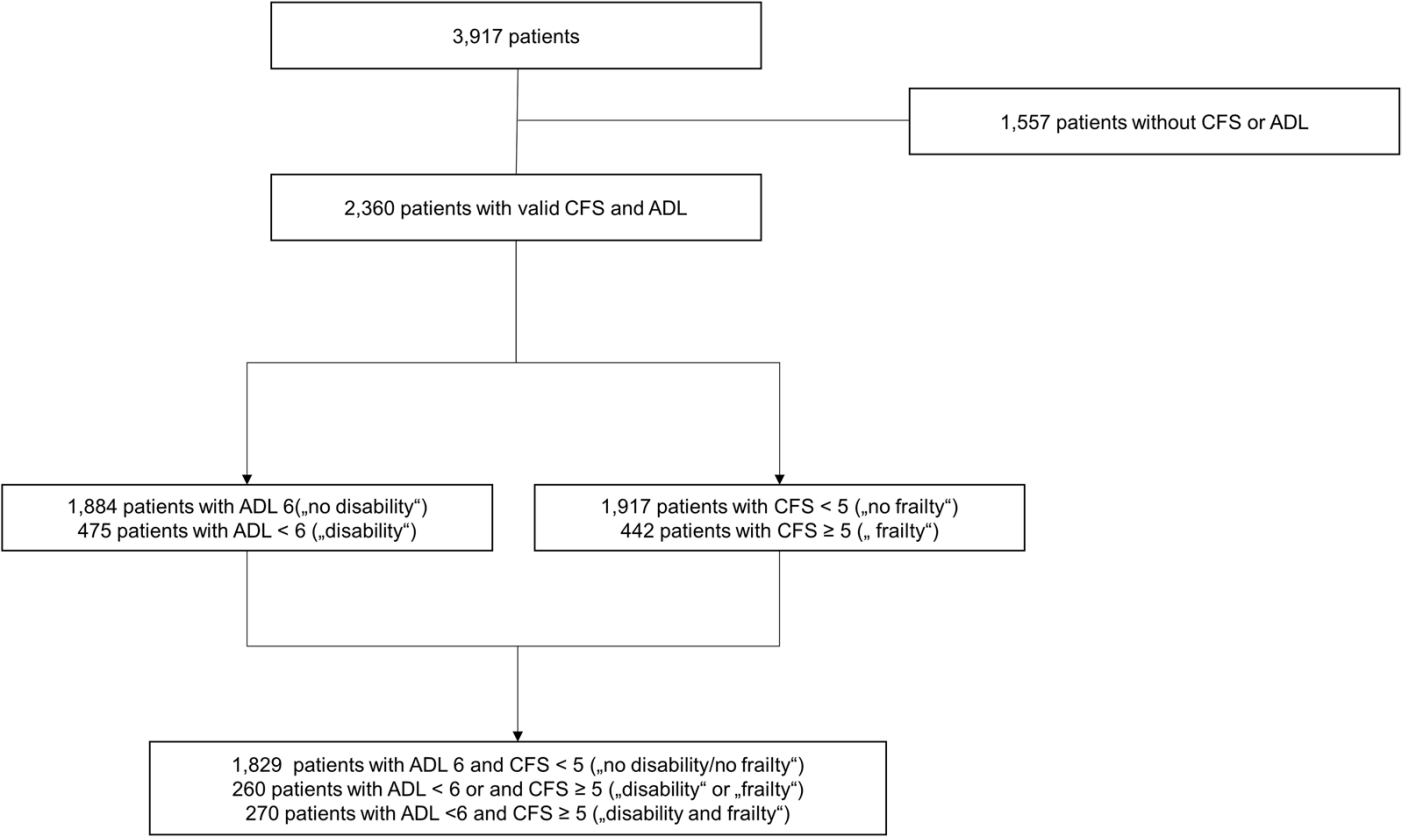
Consort diagram

**Fig. 2:**
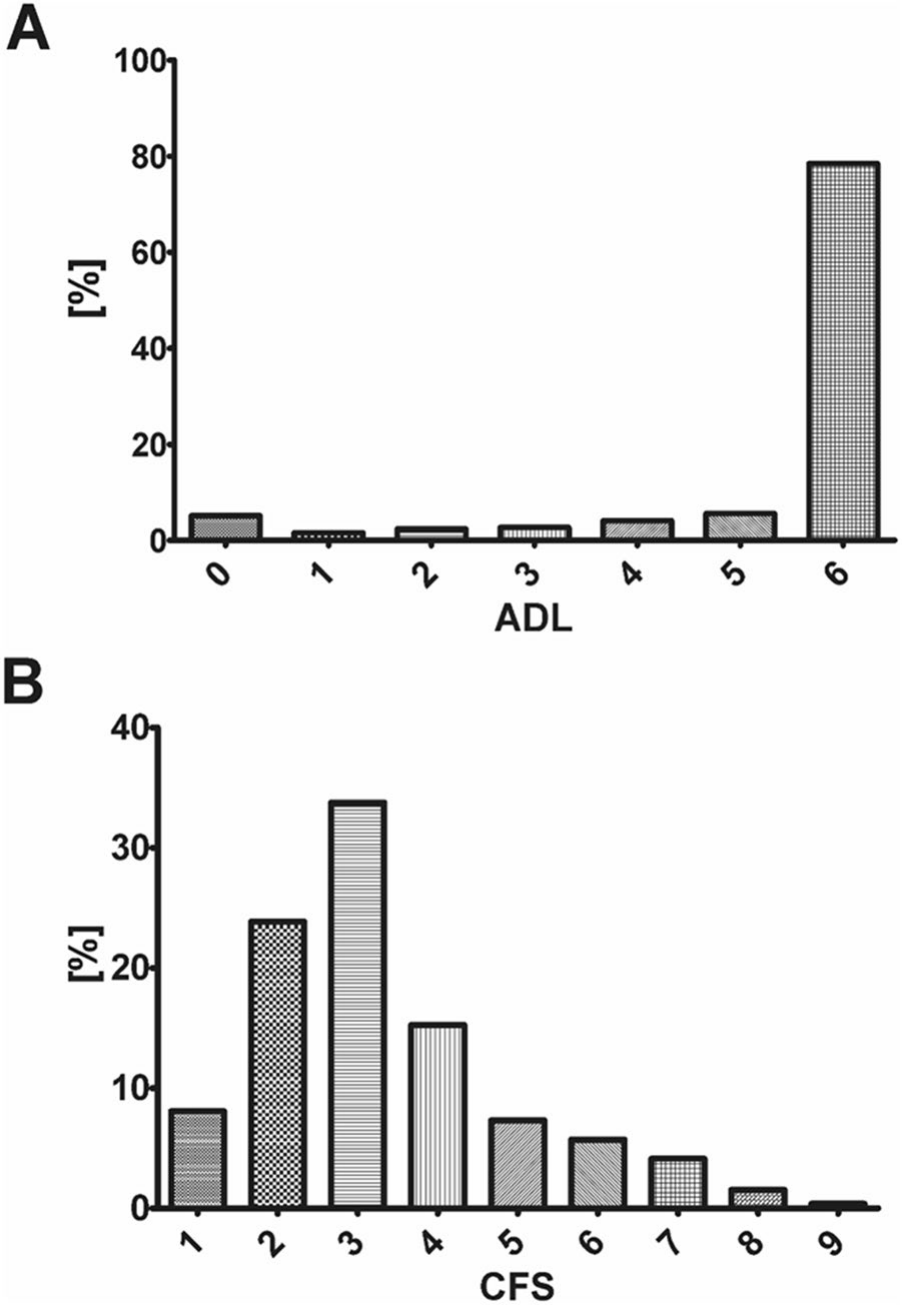
**A** Distribution of documented ADL on admission (6 = no disability; 0 = fully dependend). **B** Distribution of CFS (1 = no frailty; 9 = terminally frail)

In total, this subgroup analysis included 2359 patients from the COVIP study with a documented ADL and CFS (see Fig. 1). Most of the patients did not show any dependence in their daily living prior to hospital admission (80% ADL 6, Fig. 2A), although frailty in CFS was distributed more heterogeneously (Fig. 2B), most patients lived without severe frailty (81% CFS < 5, Fig. 2).

### Baseline characteristics of patients with disability compared to patients without disability

**Fig. 3:**
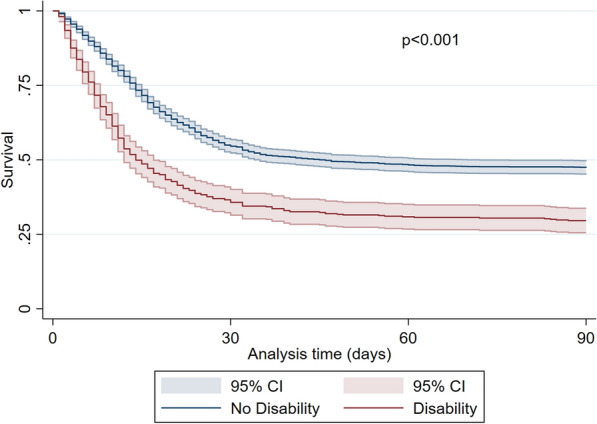
Kaplan–Meier for patients with a disability (ADL < 6, red line) compared to patients without a disability (ADL 6, blue line) (3-month mortality, ± 95% CI). *p* < 0.001 log-rank test

Patients without significant impairment in the Activities of Daily Living (ADL 6) were predominantly male (74%, *p* < 0.001), younger (75 years (IQR 4) vs. 78 years (IQR 5), *p* < 0.001), less frail (CFS 3 (IQR 1) vs. 5 (IQR 2), *p* < 0.001) and significantly less affected by comorbidities (Table 2). In contrast, with increasing disability patients were older, more frail and had significantly more comorbidities. SOFA score on admission was significantly lower in patients with high ADL (ADL 6: 5 (IQR 3); ADL < 6: 7 (IQR: 4), *p* < 0.001) (Fig. 3).

### Intensive care treatment and outcome of patients with disability compared to patients without disability

**Fig. 4:**
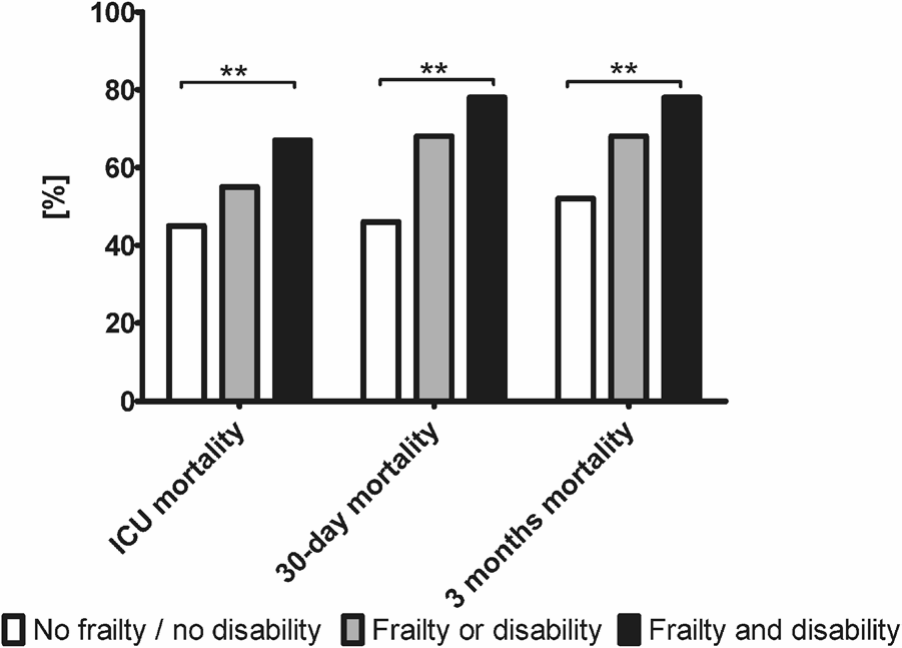
ICU-, 30-day and 3-month mortality [%] for patients with neither frailty (CFS ≤ 5) nor disability (ADL 6), frailty or disability, or frailty and disability. **p < 0.001

**Fig. 5:**
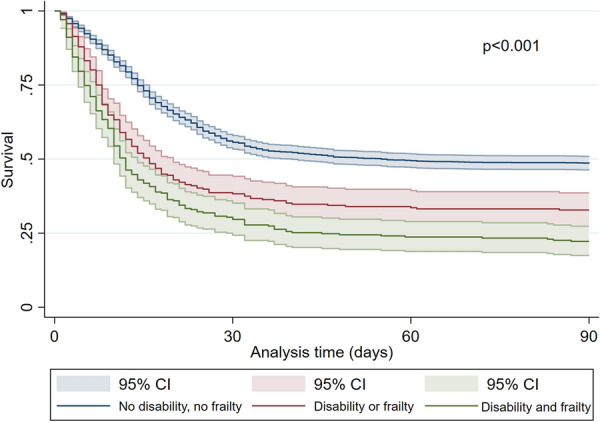
Kaplan–Meier for patients without a disability and frailty (ADL 6 and CFS < 5, blue line) compared to patients with a disability or frailty (ADL < 6 or CFS ≥ 5), red line), and patients with disability and frailty (green line) (3-month mortality, ± 95% CI). *p* < 0.001 log-rank test

**Fig. 6:**
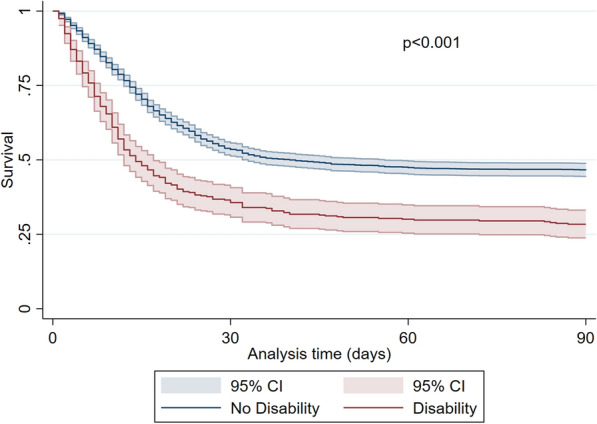
Kaplan–Meier for patients with a disability (ADL < 5, red line) compared to patients without a disability (ADL ≥ 5, blue line) (3-month mortality, ± 95% CI). *p* < 0.001 log-rank test

During intensive care treatment, patients with pre-existing disability received significantly less invasive mechanical ventilation (67 vs. 75%, p = 0.001), tracheostomy (12 vs. 20%, *p* < 0.001), vasoactive drugs (62 vs. 72%, *p* < 0.001), but more renal replacement therapies (19 vs. 14%, *p* = 0.006), and non-invasive ventilation (31 vs. 23%, *p* < 0.001). Limitations of life-sustaining therapy occurred significantly more often in patients without disability. Patients with disability suffered from significantly increased crude ICU- (62 vs. 45%, *p* < 0.001), 30-day (66 vs. 47%, *p* < 0.001), and 3-month mortality (71 vs. 53%, *p* < 0.001, Figs. 4 and 5). Using an ADL of less than 5 as cut-off resulted in similar outcomes (see Fig. 6).

In the mixed-effects Weibull proportional hazard regression, ADL was associated with 3 months mortality as a continuous variable (aHR 0.88 (0.82–0.94, *p* < 0.001)). This means that with rising ADL (= declining disability), the risk for mortality decreased. As binary variable, an ADL < 6 (“disability”) was associated with an increased 3 months mortality (aHR 1.53 (1.19–1.97, *p* 0.001), and an ADL of < 5 (aHR 1.57 (1.21–2.03, *p* 0.001), Table 3).

### Baseline characteristics of patients with frailty compared to patients without frailty

Patients with high frailty (CFS ≥ 5) were as well predominantly male (61%, *p* < 0.001), older (78 years (IQR 5), *p* < 0.001) and had significantly more comorbidities (Additional file 1: Table S1). Therefore, with decreasing frailty patients were younger and had less comorbidities. Like patients without disability, SOFA score was lower in patients with a low frailty score (CFS < 5: 5 (IQR: 3); CFS ≥ 5: 7 (IQR: 4, *p* < 0.001). After admission to the ICU, invasive mechanical ventilation (66 vs. 75%, *p* < 0.001) and tracheostomy (11 vs. 20%, *p* < 0.001) occurred significantly more often in patients without pre-existing frailty. There was no difference regarding the use of vasoactive drugs. Patients with pre-existing frailty received significantly more non-invasive ventilation (30 vs. 23%, *p* = 0.005) and more renal replacement therapies (21 vs. 14%, *p* < 0.001). Frail patients evidenced a significantly increased crude ICU- (65 vs. 45%, *p* < 0.001), 30-day (71 vs. 46%, *p* < 0.001), and 3-month mortality (77 vs. 52%, *p* < 0.001).

### Comparison of patients without disability and frailty, with disability or frailty, and of patients with frailty and disability

When dividing into the three groups no disability/frailty, either frailty or disability and frailty and disability, the results were similar: patients with frailty and disability were older (78 years (IQR 5), *p* < 0.001) and had significantly more comorbidities compared to the former groups (Table 2). Even though there was no difference in SOFA score between patients without disability and frailty and patients with disability and frailty (non-frailty, no disability: 5 (IQR: 3); frailty and disability: 8 (IQR: 4), Table 2), the study shows individually that patients with high independence in daily living evidenced lower scores of organ failure on admission and patients without frailty evidenced lower scores of organ failure on admission.

There were significant differences both in short- and long-term outcome: combining ADL and CFS to three groups (no disability/frailty, either frailty or disability, frailty and disability) resulted into the following.

The two variables frailty and disability as well as either frailty or disability were significantly associated with the 3-month mortality (Table 4): suffering from frailty or disability, was an independent risk factor (aHR 1.88 (1.47–2.40, *p* < 0.001)) but the highest risk was found for patients with both frailty and disability (aHR 1.94 (1.39–2.71, *p* < 0.001)). Patients with no frailty and no disability evidenced a significantly lower mortality (ICU-mortality 45%, 30-day mortality 46%, 3-month mortality 52%, *p* < 0.001), patients with frailty and disability the highest mortality (ICU-mortality 67%, 30-day mortality 72%, 3-month mortality 78%, *p* < 0.001, Table 3, Figs. 5 and 6). Patients who suffered either from frailty or disability were in-between (ICU-mortality 55%, 30-day mortality 63%, 3-month mortality 68%, *p* < 0.001). Therefore, with the detection of both ADL and CFS a subset of patients with an almost 80% 3 months mortality can be identified.

## Discussion

The Activities of Daily Living before acute illness have been proposed as a tool for outcome prediction in triage during the COVID-pandemic. The present study is based on the large international COVIP database and reveals that ADL is an independent prognosticator for outcome in critically ill old patients admitted to the ICU and who suffer from COVID-19. However, most patients evidenced a high degree of independence on admission, which might be interpreted as consequence of triage. It is well known that pre-existing disability is a risk factor in intensive care patients, but the crucial finding of our study is that combining of pre-existing disability with pre-existing frailty identifies a subgroup with extraordinary high mortality.

Historically, there are different approaches to assessing the functional status of old patients before ICU admission. Many studies in intensive care used functional assessments of survivors of critical illness with respiratory failure such as ADL [17–20]. There are insufficient data on what ADL scores should be considered "normal" in a selected population of critically ill and old ICU patients. Level et al. conducted a prospective cohort study with 188 patients aged 75 years or older admitted to the ICU. They found a median ADL of 4.2 ± 1.6 on admission. Furthermore, ADL on admission was independently predictive for one-year mortality [21]. Maziere et al. investigated a similar cohort with 223 critically ill, old patients. In their investigation, ADL at admission was 3.8 ± 2.2. An ADL from 0 to 3 was defined as severe dependence/disability and was significantly associated with nosocomial infection (*p* < 0.05) [22]. In a very small cohort of 16 intensive care patients, pre-admission ADL was 6 (IQR 5–6) [23].

There is no commonly accepted cut-off for ADL that distinguishes "dependence" from "independence". Giannasi et al. defined every patient with an ADL below 6 as "dependent". Their prospective cohort study included 249 patients aged 65 years or older who were admitted to the ICU and required mechanical ventilation for more than 48 h. The logistic regression analysis with adjustment for APACHEII score and age revealed an independent association of ADL with mortality (OR: 2.35, 95% CI: 1.16–4.75) [24]. Demesielle et al. used an ADL cut-off of 5 in their prospective multicentric observational cohort study with 501 patients aged 75 years or older who required mechanical ventilation. They found that an ADL ≤ 5 was not associated with increased in-hospital mortality (OR 0.88; 95% CI 0.54–1.42, *p* = 0.598), but increased 1-year mortality (aOR 0.53, 95% CI 0.30–0.96, *p* = 0.038) [11]. In 123 ICU patients with severe pneumonia, Sangla e al. defined three groups of dependence for the 1-year follow-up: ADL of 6, from 5 to 3, and below 3 [25]. Schweikert et al. defined a cut-off below 6 as dependence in a prospective interventional intensive care study in patients aged 18 years or older [26]. Langlet et al. used an ADL score of 6 to define a full function, an ADL of 5 a low degree of impairment, an ADL 4–3 for moderate impairment, and two or less for severe functional impairment. Their study compared 26 patients with chronic obstructive pulmonary disease undergoing mechanical ventilation. In their study, the ADL score was a significant predictor of 6-month mortality [12]. VIP-2 used a cut-off of an ADL of less than 5 defining “disability” [1]. In the present study, there was no relevant difference between the ADL cut-offs 5 and 4 (for ADL ≤ 5, see Fig. 6).

The timing of ADL use also differs between studies. While many use the pre-acute condition as a reference, other studies use the ADL score at the time of discharge from acute care. MacDonald et al. scored 42 patients who were discharged after being treated with veno-venous extra-corporal life support for acute respiratory failure. They found high ADL scores, indicating high independence and functionality in 62% of patients [27]. In a prospective, multicentre cohort study that recruited patients who were admitted to the ICU with respiratory failure or shock, a relevant dependence could be found in 23% of the patients 12 months after discharge. Of note, in this study, disability was defined as ADL < 6 [28].

In VIP-2, survivors had significantly higher ADL values than non-survivors (6 (5–6) vs. 6 (3–6), *p* < 0.001); 27.7% (962/3473) of the patients had an ADL ≤ 4; and 59.6% an ADL of 6 [1]. By contrast, in the present COVIP-study, only 16% (430/2692) patients had an ADL ≤ 4; and 84% had an ADL > 5 (2262/2692). However, it should be noted that VIP-2 included patients aged 80 years and older, but COVIP included patients aged 70 years and older. In the pandemic of SARS-Cov-2, CFS provides valuable and reliable information for outcome prediction [6]. Compared to CFS, the assessment of ADL might be more time-consuming [29] (compare Additional file 1: Table S2 and Fig. S1, Additional file 2).

## Limitations

This is not the first study showing that pre-existing disability is an independent risk factor for ICU outcome, but it is the first investigating its value in a selected high-risk population of critically ill old patients suffering from COVID-19. Furthermore, to our best knowledge, it is the first investigation that analyses the overlap between frailty and disability in this particularly vulnerable cohort. Our study has some methodological limitations. For example, we did not have a control group of younger COVID-19 patients for comparison or a comparable age cohort of patients who were not or could not be admitted to the ICU. In addition, COVIP does not capture information on pre-ICU care and triage. Thus, it might be hypothesised that during pandemic peaks patients with low ADL might not have been admitted to the ICU, and therefore do not appear in COVIP. Participating countries varied widely in their care structure. This results in a large degree of heterogeneity. The fact that CFS was recorded more frequently overall than the ADL is probably also due to the study design of the COVIP group. COVIP, and its predecessors, focused on the role of CFS for outcome prediction. It may be argued that there is a strong overlap between frailty and disability, so that both scales measure the same thing. However, it is argued that the mortality of the group who are both frail and dependent are significantly more at risk than patients who suffered only either from frailty or disability. For this reason, this study supports that both scales allow a complementary analysis of the patient. Last, the time frame of pre-existing ADL had not been defined in detail by the study. Thus, we do not know, if ADL reflects one month or one year before acute COVID-19.

## Conclusion

In critically ill old intensive care patients suffering from COVID-19, most patients evidenced high degrees of independence in Activities of Daily Living before ICU admission. Combining pre-existing frailty with pre-existing disability identifies a subgroup that evidences extremely high mortality rates. Thus, the initial assessment of ADL might offer an additional value for outcome prediction.

## Evidence before this study

The value of Activities of Daily Living (ADL) could be used for outcome prediction of critically ill elderly patients.

## Added value of this study

This study with 2359 patients investigated the role of ADL in outcome prediction in severe cases of COVID-19. The combination of ADL with frailty might provide additional prognostic information.

## Implications of all the available evidence

ADL offers additional information on intensive care and 3-month mortality, although most patients evidenced normal degrees of independence prior to ICU admission. The combination of an increased frailty (according to Clinical Frailty Scale) with reduced independence (= increased disability) in the Activities of Daily Living identifies a subgroup with mortality rates up to 80%.

## Supplementary Information

**Additional file 1: Table S1:** Baseline characteristics of patients with Frailty (CFS ≥ 5) and without (CFS < 5). **Table S2:** Baseline characteristics of patients who survived 3 months after ICU admission. **Figure S1**. Katz Index of Independence in Activities of Daily Living (ADL) from the COVIP-CRF, with permission.

**Additional file 2.** COVIP study group.

## Data Availability

Individual participant data that underlie the results reported in this article are available to investigators whose proposed use of the data has been approved by the COVIP steering committee. The anonymised data can be requested from the authors if required.
