# Supplementary material for: The association of the Activities of Daily Living and the outcome of old intensive care patients suffering from COVID-19
Source: Ann Intensive Care. 2022 Mar 18;12:26. doi: 10.1186/s13613-022-00996-9 (PMC8931579; doi:10.1186/s13613-022-00996-9)
Supplement: Supplementary file 1 — Additional file 1: Table S1: Baseline characteristics of patients with Frailty (CFS ≥ 5) and without (CFS < 5). Table S2: Baseline characteristics of patients who survived 3 months after ICU admission. Figure S1. Katz Index of Independence in Activities of Daily Living (ADL) from the COVIP-CRF, with permission. [file 13613_2022_996_MOESM1_ESM.docx]

**Additional file 1**

| Additional file 1 Table S1 | CFS < 5 | CFS ≥ 5 | p-value |
| --- | --- | --- | --- |
|  | N=1,917 | N=442 |  |
| Male sex ([%], n) | 73% (1,400) | 61% (270) | <0.001 |
| Age (years, ± SD) | 75 (4) | 78 (5) | <0.001 |
| SOFA | 5 (3) | 7 (4) | <0.001 |
| Diabetes mellitus ([%], n) | 32% (606) | 52% (229) | <0.001 |
| CAD ([%], n) | 20% (379) | 37% (161) | <0.001 |
| Chronic renal failure ([%], n) | 12% (224) | 33% (145) | <0.001 |
| Arterial hypertension ([%], n) | 64% (1,227) | 79% (349) | <0.001 |
| Pulmonary disease ([%], n) | 21% (398) | 34% (151) | <0.001 |
| Chronic heart failure ([%], n) | 11% (219) | 31% (133) | <0.001 |
| ADL –Activities of Daily Living; BMI – body mass index; CFS – Clinical Frailty Scale; CAD – coronary artery disease; S.D. – standard deviation; SOFA – Sequential Organ Failure Assessment; p-value comparing all groups | | | |

| Additional file 1 Table S2 | No frailty (CFS <5) /  no disability (ADL 6) | Frailty and disability | p-value |
| --- | --- | --- | --- |
|  | N=925 | N=103 |  |
| *Baseline characteristics* | | | |
| Male sex ([%], n) | 70% (650) | 63% (65) | 0.13 |
| Age (years, ± SD) | 75 (4) | 77 (5) | <0.001 |
| SOFA | 5 (3) | 6 (3) | 0.005 |
| CFS | 3 (1) | 6 (1) | <0.001 |
| Diabetes mellitus ([%], n) | 29% (267) | 55% (57) | <0.001 |
| CAD ([%], n) | 18% (167) | 39% (39) | <0.001 |
| Chronic renal failure ([%], n) | 7% (66) | 24% (24) | <0.001 |
| Arterial hypertension ([%], n) | 63% (586) | 83% (86) | <0.001 |
| Pulmonary disease ([%], n) | 20% (189) | 39% (40) | <0.001 |
| Chronic heart failure ([%], n) | 9% (86) | 32% (33) | <0.001 |
| *Intensive care treatment and outcome* | | | |
| Invasive mechanical ventilation | 66% (605) | 50% (51) | 0.001 |
| Non-invasive mechanical ventilation | 23% (215) | 28% (29) | 0.25 |
| Tracheostomy | 23% (208) | 13% (13) | 0.022 |
| Vasoactive drugs | 61% (556) | 40% (40) | <0.001 |
| RRT | 7% (66) | 13% (13) | 0.044 |
| Life sustaining care withheld | 10% (88) | 19% (20) | 0.002 |
| Life sustaining care withdrawn | 0% (1) | 0% (0) | 0.74 |

**Additional File 1 figure S1**: Katz Index of Independence in Activities of Daily living (ADL) from the COVIP-CRF, with permission.
